# Learning private equity recommitment strategies for institutional investors

**DOI:** 10.3389/frai.2023.1014317

**Published:** 2023-02-07

**Authors:** Emmanuel Kieffer, Thomas Meyer, Georges Gloukoviezoff, Hakan Lucius, Pascal Bouvry

**Affiliations:** ^1^Faculty of Science, Technology and Medicine, University of Luxembourg, Esch-sur-Alzette, Luxembourg; ^2^SimCorp Luxembourg SA, Luxembourg, Luxembourg; ^3^European Investment Bank, Luxembourg, Luxembourg

**Keywords:** private equity, evolutionary learning, recommitment strategies, artificial intelligence, optimization

## Abstract

Keeping strategic allocations at target level to maintain high exposure to private equity is a complex but essential task for investors who need to balance against the risk of default. Illiquidity and cashflow uncertainty are critical challenges especially when commitments are irrevocable. In this work, we propose to use a trustworthy and explainable A.I. approach to design recommitment strategies. Using intensive portfolios simulations and evolutionary computing, we show that efficient and dynamic recommitment strategies can be brought forth automatically.

## 1. Introduction

Over the last decades investing in private equity (PE) and real assets^1^ has gained considerable momentum as described in Cumming et al. (2022). Achieving high exposure to PE is a challenge faced by institutional investors like insurers, pension funds, banks, endowments, and sovereign wealth funds who in recent years have been building sizable allocations to this alternative investment vehicle. Successfully acquiring, managing, and exiting these investments directly demands strong expertise and incentives that most institutional investors are lacking. This is the reason why such investors generally prefer to invest indirectly as so-called limited partners (LPs) through limited partnerships funds (in the following referred to as “funds”) in which they commit a sizeable amount of capital for a given period of time.

Commitments to funds are in practice immutable, and the invested capital is called progressively by the fund's management at its sole discretion. Capital calls cannot be determined in advance which leads to committed but un-invested capital waiting to be called. Generally, the committed capital will not be drawn in full by the end of the fund lifetime. Therefore, only between 60 and 70% of the fund's commitments will really be invested in PE and yield a return in line with such assets. As the fund progresses, there will also be pay-outs from early divestments. These cash inflows must be recommitted into new funds. To make matter worse, the illiquid nature of PE stakes yields a high risk during the fund's early years when the likelihood to be called is the highest, i.e., during the investment period of the fund.

In a nutshell, the three essential aspects making PE investing so challenging for LPs are the following ones:

(1) Capital is not called in full.
(2) Committed but uncalled capital.
(3) Risk of becoming a defaulting investor.

For all these reasons, it can be very challenging to convert commitments into a stable and high exposure to private equity. In fact, an additional expertise to determine how to size and time recommitments into new funds is required to draft an efficient commitment pacing while securing liquidity for future capital calls.

As of yet, commitment pacing is tackled with portfolios models based on, despite their unpredictable nature, deterministic cashflow forecast. Usually these models are spreadsheet-based, very simple and work through “trial-and-error”. The impact of (alleged) skills when selecting high-quality funds is either not reflected or over-estimated. Often, for the targeted portfolio composition, there are no funds with desired characteristics available or not available at the time. In addition, the secondary market for funds is difficult to factor in, as it tends to dry up precisely when LPs experience liquidity problems.

Retaining the uncalled capital as dry powder remains one of the most overlooked aspect of investing in private equity (Arnold et al., 2017). However, academic research on this topic is still at its infancy. To minimize the impact of uncalled capital, LPs commit practically more capital in aggregate than available by running overcommitment strategies. They expect to fill the gap using future distributions. They also rely on the fact that the capital will not be entirely called. This simplistic strategy is a solution to (1) but increases the risk of liquidity shortage. Overcommitments share important commonalities with leverage strategies and show similar rewards and risks, notably that of becoming a defaulting investor and incurring significant financial and reputational penalties.

Recommitment strategies are essential to keep investor constantly invested at some target allocation. To the best of our knowledge, existing strategies are neither dynamic nor flexible. Heath et al. (2000) proposed to recommit the entire private equity allocation to new funds without considering past portfolios evolution. Although Nevins et al. (2004) considered distributions and commitments rates, their proposed recommitment mechanism is based on non-optimized target threshold and constant rates over time which are clearly unappropriated. Constant recommitments are not a sustainable solution in private equity as we will show later in this paper.

The seminal work of de Zwart et al. (2012) is one of the very few attempts to design dynamic recommitment strategies. Instead of relying on cashflow forecasting to solve, at each period, a single-period portfolio optimization problem, they chose to build recommitment functions relying only on current and past portfolio developments. In the same vein, Oberli (2015) extends de Zwart's work to multi-asset class portfolio including stocks and bonds. These two last attempts are strong and improving contributions to deal with the unpredictability of cashflows by generating rules to adjust dynamically the investment degree. This similarity with control theory, i.e., a domain dealing with the control of dynamical systems in engineered processes and machine, seems obvious. We aim at building a closed-loop or feedback controller to drive the system output to a desired state and ensure a level of stability. Like a physical system in which the environment can only be sensed but not predicted, the private equity controller system must actively react and compensate any deviation to the target output. Nevertheless, such a control system would have long delays and would not be allowed to over-shoot the target with the risk to create cash shortage situations.

In this work, we propose to learn such a control system, but adapted to the private equity environment. For that reason, we do not consider classical machine learning approaches even though they have been widely and successfully applied in many domains and real-world applications. Their main disadvantage is their need of supervised knowledge requiring therefore a significant amount of data which is somehow lacking in private equity. Some private market data providers generally sell data covering very specific periods and economies, but these data tend to be incomplete. There is also no guarantee for them to reflect the current market situation. We have de facto excluded deep learning methodologies which are all greedy in terms of data and computing time. Despite their strong popularity, they are also not convenient when it comes to analyze their behavior. Besides, neural networks may have millions of parameters and are often subject to overfitting. The recommitment rules proposed by de Zwart et al. (2012) are simple and very effective. What if we could generate such recommitment expressions automatically and consider that our control system would be made of such a set of strategies to be applied in specific conditions. Learning mathematical functions, i.e., symbolic expressions, is not a recent occurrence and has been widely considered by computational physicists to develop understandable models using symbolic regression. The recent advance on evolutionary learning and simulation-based optimization have paved the way to novel learning paradigms. We therefore propose hereafter a proof of concept consisting in learning symbolic mathematical expressions in the same format as proposed by de Zwart et al. (2012) and Oberli (2015) using an evolutionary algorithm (Freitas, 2003). These symbolic expressions will be then evaluated through intensive simulations to measure their performance in providing a stable and efficient control system to keep PE allocation at the target level.

## 2. The private equity recommitment problem

The PERP is a dynamic optimization problem in which an LP investor owning a portfolio is searching for recommitment levels to maintain a target allocation. PE funds have generally a lifetime ranging from 10 to 15 years. The committed capital is draw down progressively during the investment period over which the net asset value (NAV) grows continuously until it is reaching a peak around the halfway point of the fund lifetime. Once this peak has passed and fund moves into its the divestment period, the NAV along with the capital calls declines while the fund's distributions are increasing. For a single PE fund, the maximum investment level is only reached for short period of time. Consequently, it is critical to take advantage of the uncalled capital and early distributions by recommitting into new PE funds to counterbalance the opportunity cost.

To this end, the investment degree is usually considered as a metric to evaluate the fraction of the capital actually invested and is defined as:


(1) $$\begin{array}{l} ID_{t}=\frac{NAV_{t}}{NAV_{t}+Cash_{t}} \end{array}$$

**Figure 1:**
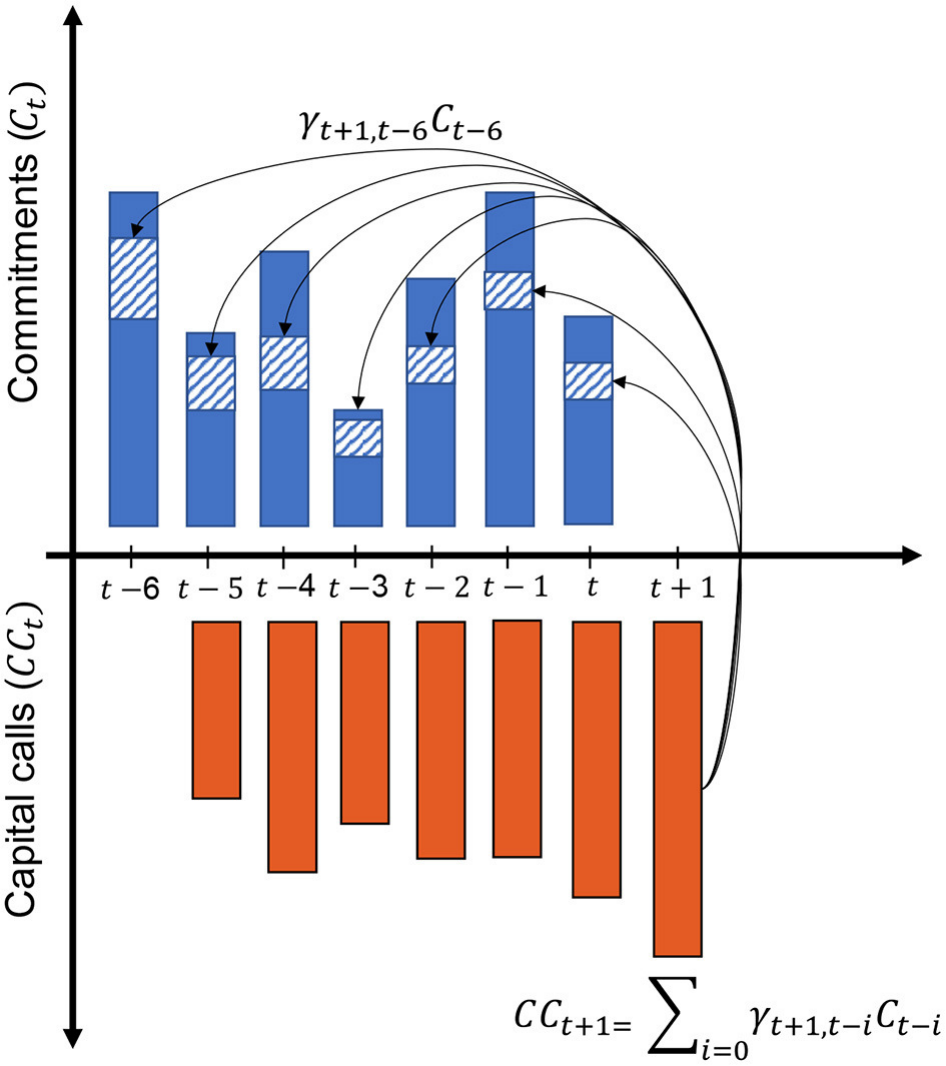
Each capital call is a fraction of the previous commitments. This fraction diminishes with time such that γ_*t*+1, *t*−*i*_≈ 0 when *i* ≥ 6 years.

The optimal amount of recommitment levels *C*_*t*_, *C*_*t*+1_, *C*_*t*+*i*_ for all periods can be theoretically obtained by solving an equivalent multi-period portfolio optimization problem defined as follows:


(2) $$\begin{array}{l} \underset{c_{t},c_{t+2},c_{t+3},\ldots }{\min}E_{t}\left(\overset{\propto }{\underset{i=1}{\sum }}{\beta^{i-1}\left(1-ID_{t+i}\right)}^{2}\right) \end{array}$$

where 0 ≤ β^*i*−1^ ≤ 1 is a discount factor, *ID*_*t*+*i*_ is the investment degree at period *t* + *i* and *E*_*t*_ is the conditional expectation at period t. This formulation is not very convenient and reflects the fact that the current optimal commitment levels depend on the future investment degree. It is therefore more appropriate to decompose the original problem into a sequence of single-period portfolio optimization sub-problems with simplified shape:


(3) $$\begin{array}{l} \;\langle \underset{c_{t}}{\min}E_{1}[{\left(1-ID_{2}\right)}^{2}],\ldots ,\underset{c_{t}}{\min}E_{t}[{\left(1-ID_{t+1}\right)}^{2}],\;\\ \underset{\;c_{N-1}}{\;\min}E_{N-1}[{\left(1-ID_{N}\right)}^{2}]\rangle \end{array}$$

where the subscript N represents explicitly the last period of commitment. Note that defining a commitment period is equivalent to have a discount factor. The analytical solution providing the optimal level of commitment *C*_*t*_ for each period, i.e., for each subproblem is thus [see de Zwart et al. (2012) for the demonstration]:


(4) $$\begin{array}{l} \begin{array}{l} C_{t}=E_{t}\left(\frac{Cash_{t}+D_{t+1}-\sum_{i=1}^{T}\gamma_{t+1,t-i}C_{t-i}}{\gamma_{t+1,0}}\right) \end{array} \end{array}$$

with γ_*t*+1, *t*−*i*_, the fraction of capital committed *i* periods ago and called at *t*+1 (see Figure 1). *D*_*t*+1_ represents future distributions at the next period. γ_*t* + 1, 0_ designates the fraction of the new commitment called immediately.

Although this sequence of sub-problems formulation gives access to an analytical solution, LP investors must forecast cashflows as well as the expected capital calls for the subsequent period. Indeed $\overset{T}{\underset{i=1}{\sum }}\gamma_{t+1,t-i}C_{t-i}\;$represents the fraction of capital from prior commitments called at *t* + 1 which is only determined by the fund manager and not known in advance.

The two main approaches documented for this purpose are:

- For cashflow forecasting see Takahashi and Alexander (2002) (also known as the “Yale model”^2^) and de Malherbe (2004).
- For engineering recommitment using strategies see de Zwart et al. (2012) and Oberli (2015).

For long-term oriented assets like PE funds there are natural limitations to any precision in cashflow forecasting. Strategies engineering is an indirect methodology aiming at approximating the analytical solution by mean of control rules. These rules do not provide an immediate optimal solution for each period but adjust recommitments until the system reached its target allocation and remains stable. They are very convenient but can be tedious to discover.

For this purpose, we suggest a novel approach based on simulation and evolutionary learning to discover them automatically. Contrary to classical machine learning which attempts to build a model from historical cashflows, we adopt a different perspective in which we consider an augmented version of the proven Yale model, to generate cashflows data with specific properties. These synthetic cashflows described hereafter leverage new opportunities to create and observe market situations that may have never existed thus far.

## 3. Building synthetic cashflows—The Yale Plus model

PE funds are, notwithstanding the emergence of a secondary market in recent years, highly illiquid. From the LP perspective, they are cashflow assets, described here in absence of a common definition as assets that cannot be traded profitably, create cashflows, and need to be sustained through timely provision of liquidity. LPs are mainly exposed to the extreme uncertainty regarding the timing and amount of their funds' capital calls and disbursements. The LPs' problem is how to model the cashflows for portfolio and risk management purposes.

One well documented technique is to consider so-called “cashflow libraries”. These are historical funds' cashflow datasets that are argued to reflect the “true” behavior of funds and thus capture the dynamics of private equity and real assets best. When forecasting for a given fund, its future development is simulated by randomly picking cashflows from this library with adjustments for the fund's strategy and the stage in its lifecycle. This technique arguably is the “gold standard” for cashflow forecasting.^3^ It combines simplicity and robustness of approach with the ability to capture the ups and downs in private markets.

Collecting a comprehensive and up-to-date data set is a cumbersome and expensive process. Larger fund-of-funds players enviously protect the rich histories in their data warehouses—with several thousand mature fund cashflows—that allow them to credibly simulate future cashflows of portfolios of funds. Some private market data providers sell such fund level cashflow data, but only for high subscription fees and often just aggregated figures for groups of funds. For the majority of LPs the cashflow library approach arguably is not a viable option.

This work considers the use of synthetic fund cashflows as a more practical solution. These cashflows are artificially generated by expanding the Yale model.

### 3.1. The Yale model

The Yale University's endowment has been investing in private equity since 1973. Their method for modeling illiquid asset funds has been described in Takahashi and Alexander (2002).

A robust and tried-and-tested approach.

**Figure 2:**
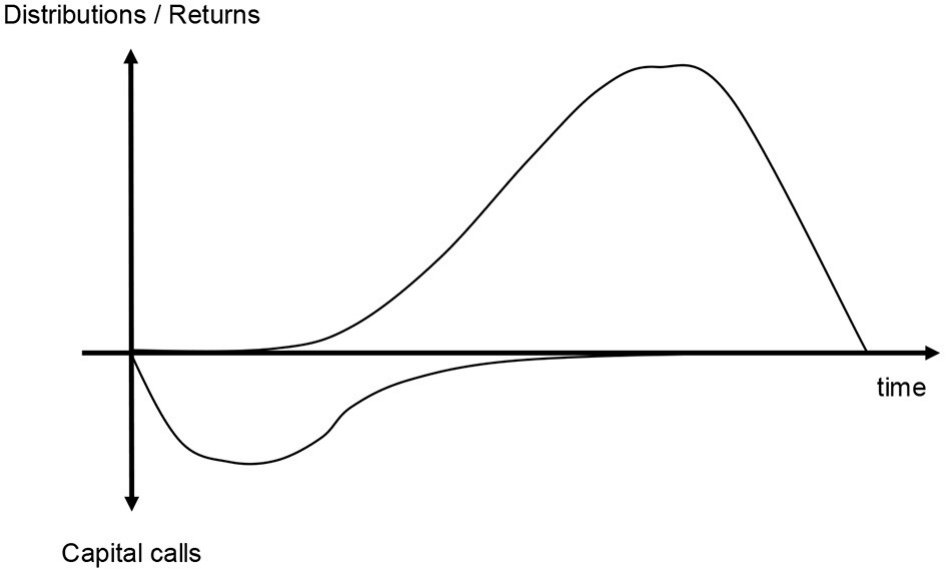
Expected cashflows projected by Takahashi-Alexander model.

This Yale model can be applied to private equity and real asset funds. It mirrors the limited partnership's actual investment cycle, distinguishing between contributions (cash inflows), distributions (cash outflows) and the NAV representing the fund's underlying assets. The timing of all cash flows, as well as the return on the committed capital, is modeled as deterministic, i.e., in contrast to a probabilistic model, a single run of this model creates just one result (i.e., expected contributions, expected distributions, and expected NAVs) for one set of input parameters and not a range of outcomes (Figure 2). Nevertheless, according to Takahashi and Alexander (2002), the projections generated fit historical data surprisingly well. In fact, the model uses the best available information for each step, e.g., contributions are projected based on the undrawn commitment for the year and the remaining distributions are based on, among other factors, the current valuation.

The major advantage of the Yale model is its simplicity and its power of explanation. It is easy to understand and allows users to identify the causal relationship between input parameters and outcomes. Being simple and sensible on a theoretical basis was one of the model's stated design objectives. Simplistic as it appears, this Yale model has been proven as difficult to beat and stacks up well against other apparently more sophisticated approaches (see Furenstam and Forsell, 2018).

#### 3.1.1. Applications

Input parameters for the Yale model are the fund's capital commitment, its lifetime in years, yearly rates of contributions, the fund's annual growth rate (%, effectively its IRR), and a so-called “bow factor” that describes changes in the rate of distributions over time. For income generating asset types such as real estate the yield sets the minimum distribution level. Yield can be interest, rental payments, but it can also describe depleting assets such as oil and gas. No paper is known to the author that refers to the use of the yield parameter. Probably more traditional techniques for modeling debt instruments are better suited for this case. As output the model projects the fund's annual capital contributions, distributions, and NAVs.

#### 3.1.2. Variations

Variations of the Yale model were presented by Hoek (2007), Tolkamp (2007), and Kocis et al. (2009). All these models, however, also create just one result for one set of input parameters and not a range of outcomes. Clearly and like with all other models, the longer the time horizon the less precise the Yale model's projections can be. While their mechanics are simple and allow going through various scenarios by adjusting input parameters, the need to estimate these parameters reduces the usefulness of non-probabilistic models. Particularly the inability to project widening ranges clearly puts limits to the use of the Yale model and its extensions for risk management purposes.

### 3.2. Stochastic expansion—The Yale Plus model

Instead of depending on the difficult task to acquire historic cashflows from comparable funds, using synthetic, i.e., artificially generated, fund cashflows can be an often more practical solution. These synthetic fund cashflows are created by funneling data generated by the deterministic Yale model through a noise-adding algorithm to construct a new data set. The resulting data set shows the statistical features and the useful patterns needed for capturing for the liquidity risks associated with portfolio of funds.

#### 3.2.1. The Yale model as starting point

**Figure 3:**
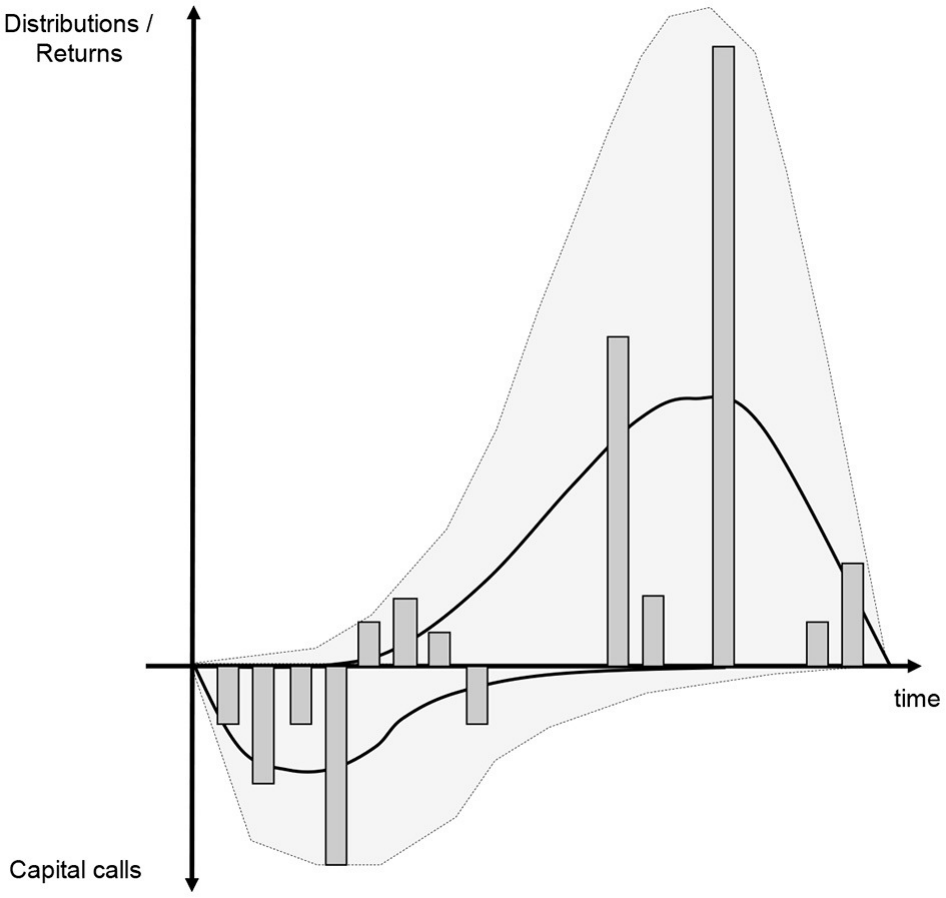
Stochastic expansion of Takahashi Alexander model.

The Yale model's main weakness is that the output quality obviously depends on how well input parameters are chosen. In its simple form it also does not offer an intuitive linkage to the market's dynamics and its uncertainty. Deterministic models do not reproduce the erratic nature of real-world fund cash flows. This may still work well for the Yale endowment with its highly diversified portfolio of funds, but investors with relatively concentrated portfolios should be aware that individual fund cashflows may vary widely and therefore returns are substantially dispersed. Under these circumstances any deterministic model does not sufficiently address risk measurement concerns. Notably the Yale model does not capture the extremes and the volatility in regards of timing and amounts of cashflows (Figure 3).

The Yale plus model builds on the Yale model but projects randomly distributed cashflows. It also estimates a NAV that is consistent with these cashflows.

#### 3.2.2. Changing the periodicity of forecasts

The first important extension is by allowing for different periodicities, i.e., not only yearly forecasts but also semi-annually, quarterly, monthly. Forecasts are often quarterly as this is also in line with the frequency of the investment reporting in the private industry. At least for short-term projections LPs may also need monthly forecasts.

For yearly deterministic forecasts it makes sense just to consider two types of cashflows, i.e., contributions and distributions. For a stochastic model and other periodicities, we need to differentiate between cashflows that remain largely deterministic and cashflows that are more random in nature. Therefore, the Yale plus model forecasts four different types of cashflows: drawdowns, management fees, repayments and fixed returns. Contributions are the sum between draw downs and management fees while distributions are repayments increased by fixed returns.

For this purpose, additional parameters are required to model management fees and fixed returns:

- The usual calculation basis for management fees is either committed capital or invested capital.
- The investment period defines until when management fees are to be calculated based on the committed capital. Once the investment period expires, the vast majority of fund managers begin to receive a discounted management fee.
- The Yale model's yield parameter is extended by being able to set its frequency and the first period of when the yield is generated as yield schedule.^4^

#### 3.2.3. Injecting randomness

In order to capture the volatility of cashflows we need to model (1) how many cashflows take place within the year, (2) how cashflows are allocated to the respective period, and (3) how the amounts of cash-flow are distributed within the period. The Yale plus model therefore requires the following additional parameters to model the randomness of cashflows:

- The frequency of drawdowns within the investment period and after the investment period (typically reduced frequency).
- The volatility of drawdowns within the investment period and after the investment period (typically reduced frequency).
- The frequency of repayments within the investment period (typically reduced frequency) and after the investment period.
- The volatility of repayments within the investment period (typically reduced frequency) and after the investment period.

The Yale plus model produces randomly distributed cashflows that are not correlated between the different periods.

#### 3.2.4. Determining a cashflow consistent NAV

The Yale model is setting a deterministic relationship between contributions, NAVs, and distributions. This relationship does not hold any longer for randomly distributed cashflows. The Yale plus model, however, applies the same logic to estimate a NAV that is consistent with the fund's cashflows. Like in the Yale model it is assumed that the underlying portfolio (captured in the NAV) is growing with the rate given by the fund's IRR. Funds tend to have mainly contributions in the beginning of their life and mainly distributions at the end of their lifetime. There is some fuzzy-ness during the fund's mid-life, where there are contributions as well as distributions within the same period. However, during this phase the NAV tends to show its maximum, so that it is unlikely that distributions exceed the available NAV. Note that this is the NAV attributable to the LPs, as the Yale plus model, like the Yale model, projects repayments on a net basis.^5^

### 3.3. Results

**Figure 4:**
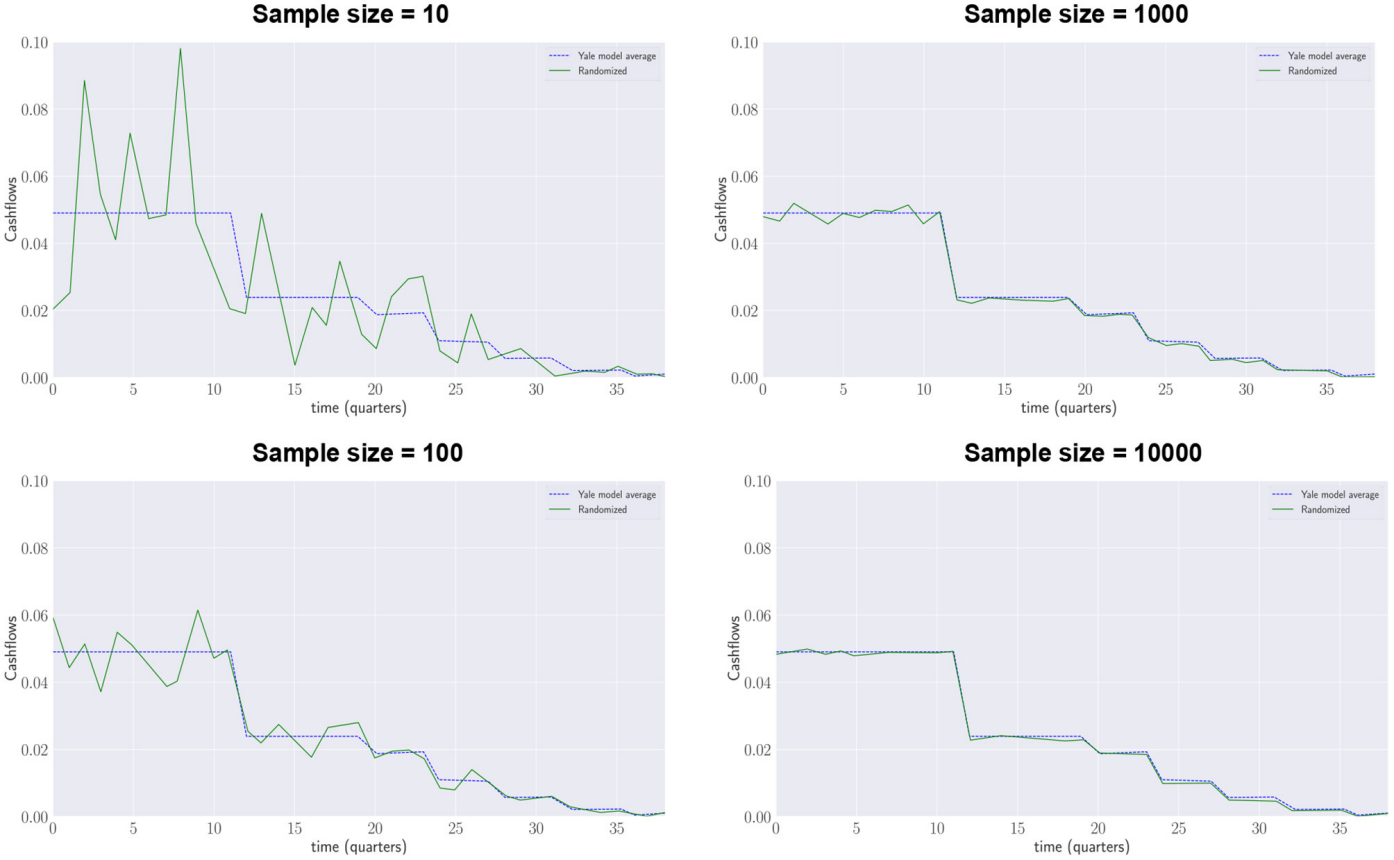
Yale Plus model, quarterly drawdowns.

The Yale plus-model aims to be a generalization of the Yale model: its average projected annual contributions and distributions have to be the same as those projected by the deterministic Yale model. Figure 4 shows how with an increasing number of samples generated by the Yale plus model (green lines) the results converge to the contributions forecasted by the deterministic Yale model (blue dotted lines).

**Figure 5:**
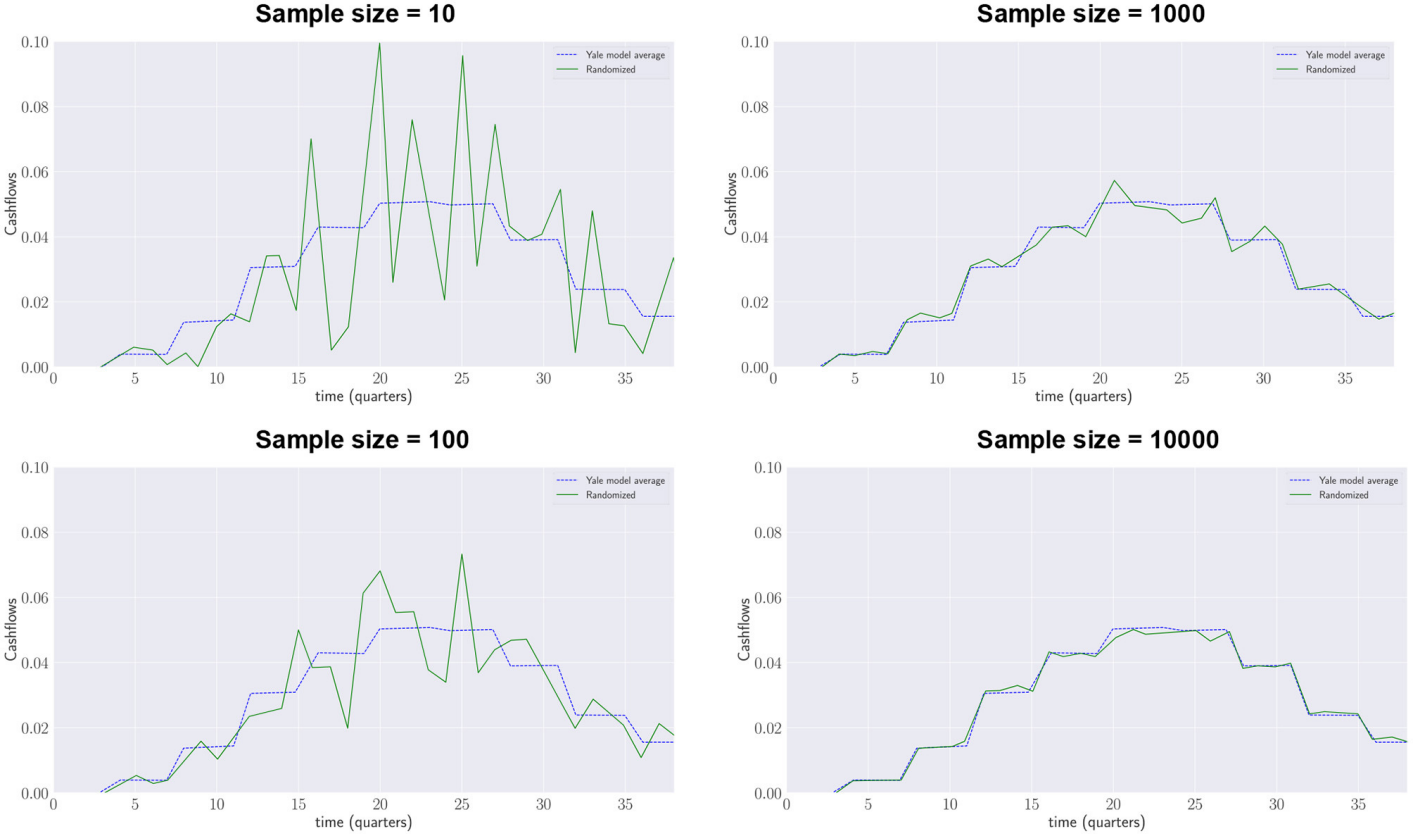
Yale Plus model, quarterly repayments.

Figure 5 shows how with an increasing number of samples generated by the Yale plus model (green lines) the results converge to the distributions forecasted by the deterministic Yale model (blue dotted lines).^6^

The Yale plus model describes the funds' typical behavior unbiased by the market. Various stresses can be applied on the forecasted cashflows without the risk of double counting.

Obviously, the Yale plus-model can generate as many cashflow samples as needed. In case that there is a significant exposure to particular situations such as venture capital funds or emerging markets, more specific models could be beneficial as building blocks. Data observed for these different fund strategies can be used to calibrate the Yale plus model for setting the required parameters. Alternatively, they can be set by expert, for instance in situations where there are no of insignificant historical data available.

## 4. Learning recommitment strategies with evolutionary learning

The last decade has seen an exponential growth in the artificial intelligence field of study. The literature is replete with numerous innovations in terms of knowledge discovery (KD) going from computer vision (see Krizhevsky et al., 2017) to natural language processing (Devlin et al., 2019). The last wave in AI research and notably in Deep Networks has reached a level of efficiency that has never been experienced before. AI-based algorithms are now challenging Human performance in many domains. The Go game invented 2,500 years ago in China remained one of the few games where Human players were undefeated until “Google's AlphaGo beats Humanity” in 2016.

Using a library of synthetic cashflows generated from the Yale Plus model discussed in the previous section, we attempt to train recommitment strategies to maintain a target investment degree as close as possible to the ideal one while ensuring that future capital calls can be satisfied. This will be achieved using simulation-based learning and, more precisely, by exploiting Evolutionary Learning, i.e., a Bio-inspired Artificial Intelligent class of algorithms.

### 4.1. Learning using Darwinian principles

Evolutionary Learning addresses optimization problems in machine learning using evolutionary algorithms (EAs) which are stochastic bio-inspired search algorithms relying on Darwinian evolution. This family of global optimization algorithms (see Zhang and Xing, 2017; Katoch et al., 2020) is well-suited for complex mathematical problems, such as non-differentiable, dynamic, non-convex and multi-objective functions which practitioners may encounter in daily business.

Recently, Evolutionary Learning approaches have been experiencing a renewed of interest, especially with the development of so-called hyper-heuristics algorithms (see Drake et al., 2020). Hyper-heuristics algorithms belong to the class of “Learning to optimize” approaches in which heuristics, i.e., rules of thumb and educated guesses, are generated through a learning process. These approaches do not only focus on discovering a single solution but also on providing the mean to get to the solution. A famous quote says: “*Give a Man a Fish, and You Feed Him for a Day. Teach a Man To Fish, and You Feed Him for a Lifetime*”. This is clearly the main philosophy of hyper-heuristics variants found in the literature. Considered as “off-the-peg” approaches by Burke et al. (2013) as opposed to “made-to-measure”, the development of hyper-heuristics expresses a need of generalization to automatically design heuristics or simply “frugal” rules to tackle problems. Decision makers and domain experts generally prefer simple and trustworthy approaches to take decisions. Unfortunately, and despite their success in the academic world, outcomes provided by deep neural networks are still very difficult to analyse on real-world applications. For this kind of applications, trust is as valuable as performance.

This is the reason why learning intelligible rules can be very helpful for investors in private equity who wish to better grasp results provided by Artificial Intelligent algorithms.

### 4.2. Recommitment strategies as heuristics

Recommitment strategies as defined by de Zwart et al. (2012) are mathematical functions computing the amount of capital that should be recommitted during the current period *C*_*t*_. There are therefore symbolic expressions which can be represented as pieces of a program.

Training symbolic expressions has been extensively considered for regression as pointed out in Žegklitz and Pošík (2020). The objective is to work with a generic and non-parametric model to fit data while being freed from the burden of choosing the best regression algorithms. Discovering symbolic expression has been easily extended to general optimization problem, especially combinatorial problems which are all belonging to the NP-hard class of optimization algorithms. The literature is replete with hyper-heuristics relying on genetic programming (GP) in which symbolic expressions modeled as abstract syntax trees (ASTs) are evolved using Darwinian principles to discover promising optimization rules. The suitability of GP algorithms has been established by Fukunaga (2004) for the well-known SAT problem. Theses algorithms have the major advantage to automatize the assembly of the components required to create a heuristic. Few investigations [in Finance (Kampouridis et al., 2013)] have been undertaken to apply heuristic generation using GP algorithms in Finance while it has notably encountered real successes in combinatorial optimization problems (Sabar et al., 2013; Sabar, 2015) and more specifically in cutting and packing (Burke et al., 2012), scheduling (Branke et al., 2016) and other additional domains such as function optimization (Oltean, 2005), real-time logistics (van Lon et al., 2012).

### 4.3. Evolution of Recommitment Strategies

**Figure 6:**
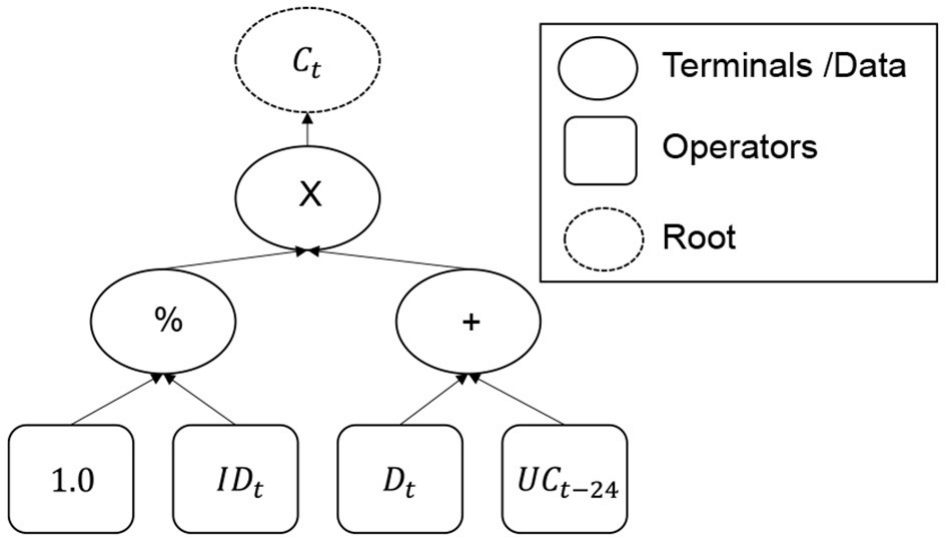
Operators and terminals.

As aforementioned, strategies are mathematical expressions. They can be represented as symbolic expressions through abstract syntax trees (see Figure 6) composed of 3 kinds of nodes:

- **The root** which represents the amount of capital to be recommitted.
- **Operators** which stand for mathematical operators.
- **Terminals** representing data obtained from simulated portfolios.

Multiple trees can be combined and altered to discover new promising strategies. For this purpose, we rely on a classical GP algorithm which borrows the concept of “Natural Selection” from Darwin to drive an initial population of randomly generated recommitment strategies to develop appropriate features for near-optimal recommitted capital. Learning is therefore achieved by mean of generational replacement in such a way that strategies with traits that enable them to maintain a target PE exposure, i.e., a target investment degree, will survive through generations and provide better altered versions inheriting those traits.

Following the Darwinian's principles, the genetic material of a strategy is defined as its tree representation which can be altered using two main operators known as “crossover” and “mutation”. These operators modify their structure by recombining, cutting or adding new operators or terminals. In this spirit, the crossover operator exhanges portions or sub-trees to mimic mating which leads to an exchange of genetic material. On the contrary, the mutation operator reflects rare but impactfull events altering randomly the genetic material.

The parallel with species evolution is the foundation of genetic programming which simulates a long-lasting natural process which gave birth to all living entities on Earth. The fittest strategies, i.e., the ones allowing to maintaing a high exposure, have more chance to be selected, propagated to the next generation. Yet again, this mechanism biaised the evolution toward strategies developing properties helping them to master their environment, i.e., private equity portfolio.

However, that is where the parallel with Darwin's evolution of species ends as we now need to provide a mathematical definition of fitness which is far from being bio-inspired. Nonetheless, interesting readers may refer to Whitley and Sutton (2012) for more details. In addtion, Table 1 is a summary codex between the key element in Darwinian Evolution and the bio-inspired optimization approach implemented in this work.

**Table 1:** Parallel with Darwinian evolution.

**Species evolution**	**Genetic programming**
Entity	Strategy
DNA	Abstract syntax tree
Breeding	Crossover operator
Mutation	Mutation operator
Survival	Fitness selection, i.e., investment degree

The search for improving strategies is performed according to the pseudocode described by Algorithm 1. At each generation, a population of NPOP recommitment strategies will be **evaluated** using simulations on a set of 250 initial portfolios, i.e., the training set. Evolutionary operators are applied on candidate strategies selected with regards to a bi-objective function representing t he deviation to the ideal investment degree and the liquidity risk. Recommitment strategies are then selected according to a Pareto rank obtained after assigning a crowding distance as performed in the NSGAII algorithm proposed by Deb et al. (2000).

**Algorithm 1:** Bi-objective Evolutionary Learning algorithm.

1:	population ← gen ramped half and half(NPOP,min,max)
2:	**for** strategy in population **do**
3:	strategy.fitness ← simulate(strategy,
	training instances)
4:	**end for**
5:	sortNonDominated(population)
6:	assignCrowdingDistance(population)
7:	**while** gen ≤ NGEN **do**
8:	parents ← selection(population)
9:	offsprings ← Ø
10:	**for** candidate strategy in parents **do**
11:	**if** random() ≤ CXPB **then**
12:	mate ← sample(parents,1)
13:	offspring1,offspring2 ← CX(candidate
	strategy,mate)
14:	offsprings ← offsprings ∪ {offspring1,
	offspring2}
15:	**else if** random() ≤ CXPB+MUTPB **then**
16:	mutant ← MUT(candidate strategy)
17:	offsprings ← offsprings ∪ mutant
18:	**else**
19:	repro strategy ← copy(candidate strategy)
20:	offsprings ← offsprings ∪ {repro strategy}
21:	**end if**
22:	**end for**
23:	**for** new strategy in offsprings **do**
24:	new strategy.fitness ← simulate
	(new_strategy, training instances)
25:	**end for**
26:	sortNonDominated(offsprings)
27:	assignCrowdingDistance(offsprings)
28:	population ← selection(population
	+ offsprings,NPOP)
29:	**end while**
30:	return population

Once evolution reached the maximum number of generations (NGEN), the best non-dominated population of recommitment strategies is returned and then finally scored with a simulation on a validation set of 1,000 initial portfolios. Naturally, both training and validation set contain different portfolios. Only the results on the validation set will be reported as it is done in classical machine learning scheme to show generalization capabilities of the resulting strategies.

### 4.4. Simulation and evaluation of recommitment strategies

Recommitment strategies will be evaluated using simulation-based learning relying on the synthetic cashflows described upstream. Nonetheless, the nature of recommitment strategies requires mature portfolios with existing investments. Therefore, we adopt the same portfolio inception and simulation protocol defined in de Zwart et al. (2012). Initial PE portfolios are constructed over a year by investing uniformly into 16 randomly selected PE funds.

**Figure 7:**
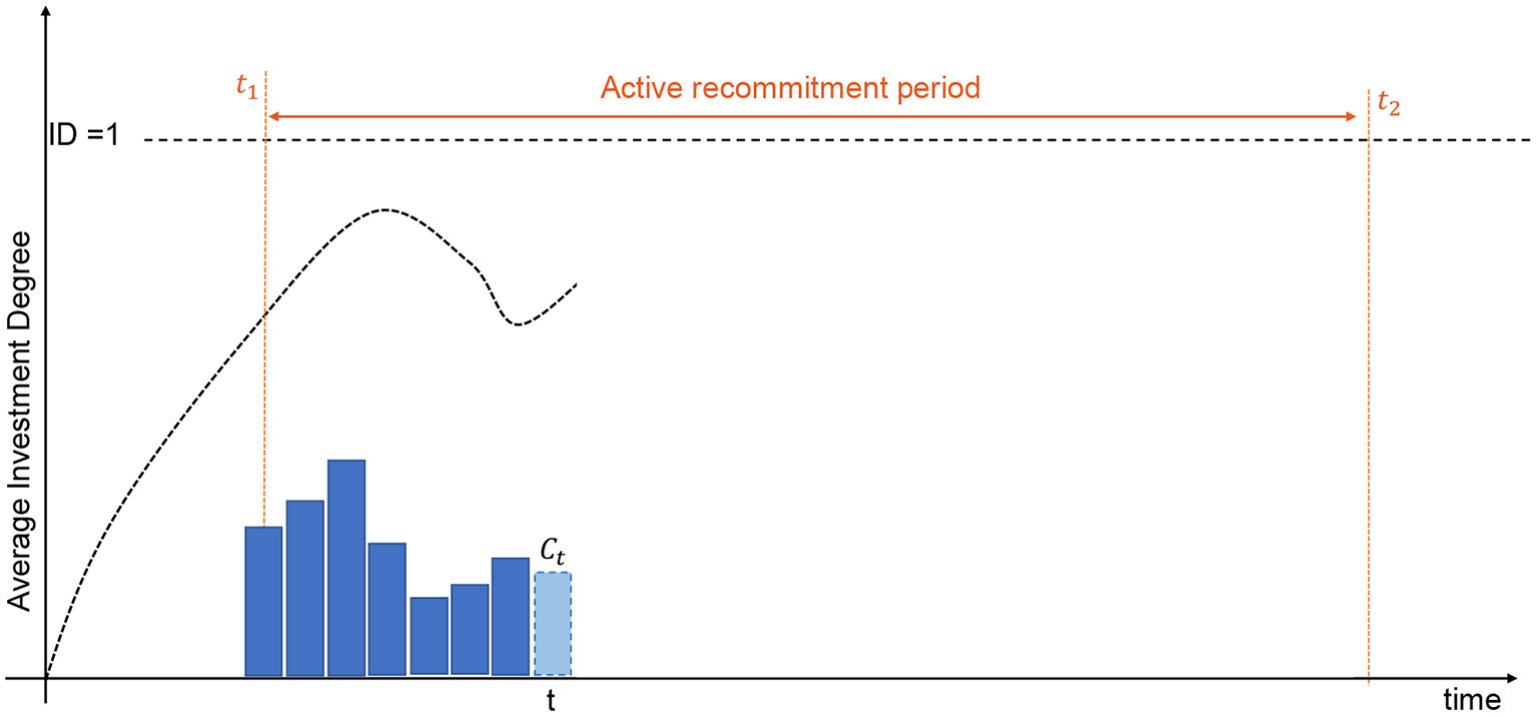
Simulation of private equity portfolios.

This set of initial portfolios represents the training set on which recommitment strategies will be learnt. A simulation just consists in recommitting some capital to new funds at every quarter for all training portfolios to obtain statistical confidence regarding the performance of the strategy. The average investment degree is then returned once the end of the active recommitment period has been reached (see Figure 7). At each period, the amount of capital is computed using the recommitment strategy currently evaluated. Once the recommitted capital has been determined, it is equally divided and invested into 4 randomly selected funds.

**Figure 8:**
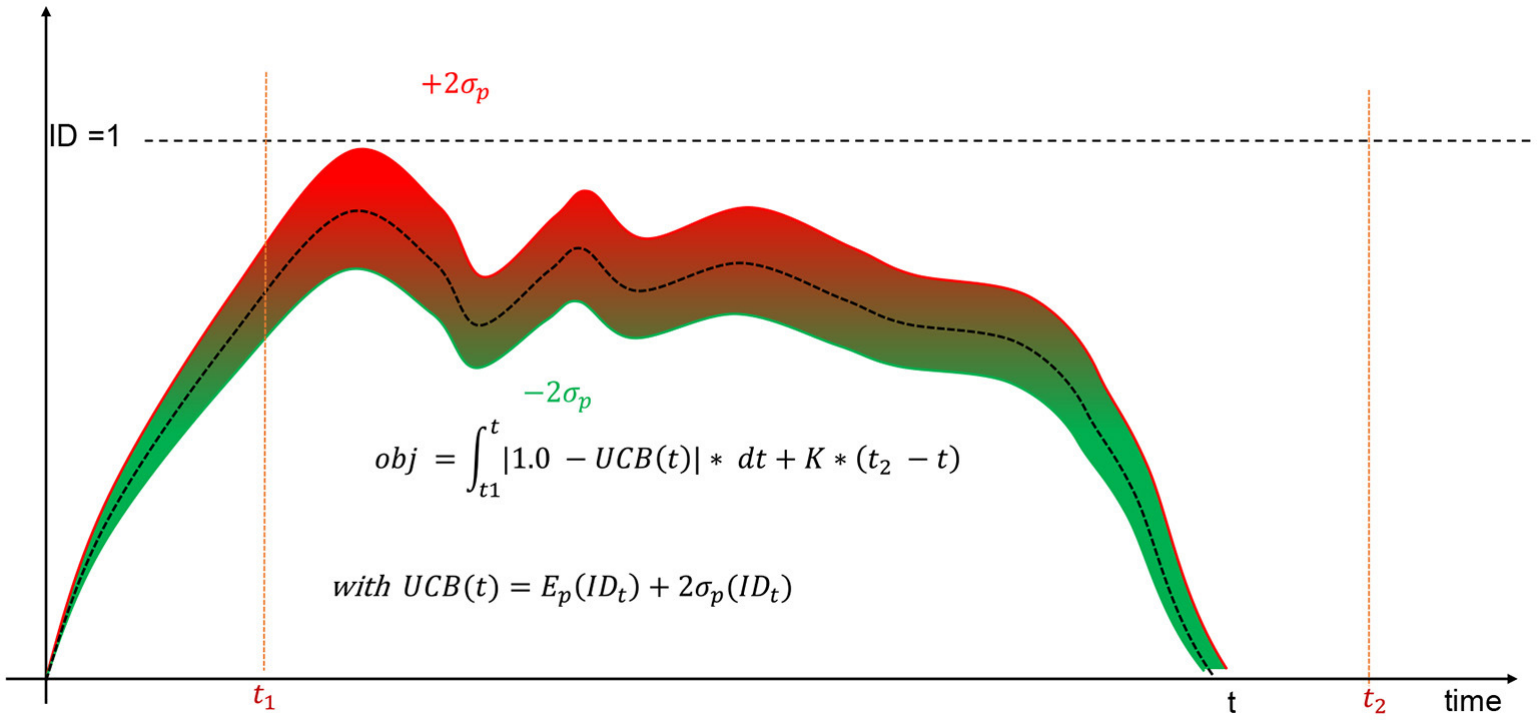
Upper-confidence bound objective.

Simulations ends when all investments have been exited which necessarily occur after *T* = *t*_2_−*t*_1_ recommitments, i.e., after the active recommitment period. Nonetheless, the fitness of the current strategy under evaluation is computed only for the active recommitment period which is stable and not subject to undue influence of the initial portfolio creation. Contrary to de Zwart et al. (2012), we do not consider the average investment degree as the only metric to measure the efficiency of a strategy. Driving the investment degree as close as possible to the ideal one will lead to situations in which some simulated portfolios will be overinvested due to cashflow variability. This is the reason all strategies should be evaluated in term of accuracy and dispersion of the investment degrees. In Kieffer et al. (2021), a preliminary investigation has considered a scalarization approach to combine both metrics into a single objective function (see Figure 8). The Upper-Confidence Bound (UCB) replaced the simplistic average investment degree considered by de Zwart et al. (2012). The objective drives only the upper bound of the 95% confidence interval to the ideal investment degree. Note also that portfolios exiting investments before the end of the active recommitment period due to ill-formed strategies are automatically penalized with a constant *K*.

Although the previous scalarization approach provided promising results, one may wonder if another strategy relying rather on a bi-objective approach would be more suitable. Indeed, investors and, more especially institutional investors, may have a different level of acceptance to the liquidity risk. This is all the more true as their portfolios are diversified with multi-class assets that are more or less liquid. In this context, the liquidity constraint described in de Zwart et al. (2012) could be modeled as an additional objective which should be minimized. Indeed, both opportunity cost and liquidity risk are two conflicting objectives. If cash is missing, some more liquid assets could be sold to cope with such a situation. Although this last solution should be tempered and only occur when no valid alternative may be found, selling liquid assets still remains less critical than becoming a defaulting investor. This is the reason why this current work focuses on two objectives, namely the deviation to the ideal investment degree and the liquidity risk, to discover a set of non-dominated recommitted strategies.

## 5. Numerical experiments

The experiments presented hereafter were carried out using the Luxembourg's supercomputer: Meluxina. The Python library DEAP has been considered for the genetic programming implementation. A distributed implementation relying on a master-slave model has been put in place to evaluate each strategy during the training phase.

### 5.1. Setups

Table 2 represents the different atomic elements constituting the future recommitment strategies. Note that the strategy n°3 published in de Zwart et al. (2012) has been embedded into the terminal set. This is an additional advantage 5.2 of genetic programming as it can embed and build on existing knowledge providing by experts.

**Table 2:** Operators and terminals.

	**Name**	**Description**
Operators	+	Add two inputs
	–	Substract two inputs
	*	Multiply two inputs
	%	Divide two inputs with protection
	Min	Minimum b.t.w two inputs
	Max	Maximum b.t.w two inputs
Terminals	*CC*_*t*_	Contributions/capital call at *t*
	*D*_*t*_	Distributions at *t*
	*ID*_*t*_	Investment degree at *t*
	*Cash*_*t*_	Remaining portfolio cash at *t*
	*NAV*_*t*_	Net asset value at *t*
	*error*_*t*_	Deviation to ideal *ID*_*t*_ at *t*
	*DZ*^3^(*t*)	De Zwart's strategy no. 3 at *t*
	*UC*_*t*−24_	Uncalled capital for commitments made 24 quarters ago
	*Ccommit*_*t*−24_	Capital committed 24 quarters ago

Table 3 lists all simulation parameters for training and validation which are aligned with the ones described in de Zwart et al. (2012). Machine learning requires the use of two different sets of initial portfolios to fairly measure the ability of the future recommitment strategies to improve the investment degree in unseen situations. Learning will be performed using the training portfolios while all reported results will be provided on the validation portfolios.

**Table 3:** Evolutionary learning parameters.

**Parameters**	**Value**
Runs	30
Generations	15
Population of strategies	1,000
Crossover operator/probability	One point crossover/0.85
Mutation operator/probability	Grow/0.1
Selection operator	Binary tournament
Tree initialization	Ramped half-and-half
Height limitation	5

We used the same simulation protocol and parameters as described page 89 in de Zwart et al. (2012). We recommit into 4 funds with synthetic cashflows. Recommitments are performed quarterly during the active recommitment period which last 26 years. Active strategies to select the best funds is out of the scope of our study. Nonetheless, the rise of Environmental, Social, and Governance (ESG) factors has been one of the major changes for investors in private equity. ESG considerations have redesigned the standards of due diligence and add new objectives on top of financial statements and growth plans. From the regulatory point of view, the landscape of ESG may seem uncertain and is part of the challenges faced by private equity investors. ESG criteria are wide and depend strongly on the underlying private equity firms and their application domains. Rules and regulations fluctuate regularly from country to country and evolve quite rapidly with the release of new studies. It is therefore not trivial to evaluate them with a single criterion adding another level of difficulties for investors who needs to deal with fuzziness and conflicting objectives. Currently, there is no automated and optimized solutions to help investors to maximize their allocation to ESG. This is the reason why the selection of funds (Table 3) is based on an artificial ESG scoring which has been computed with a specific correlation to the Total Value Paid In (TVPI). Our objective was not to design an ESG scoring mechanism which is a challenge on its own but to enable future research to combine it with the recommitment system proposed in this work. Consequently, we added to our implementation a feature enabling the use of ESG scores.

Finally, Table 4 depicts the parameters considered for the genetic programming implementation. The choice of these parameters has not been obtained through parameter tuning but based on our experience and empirical trials. We provide them for the sake of reproducibility. The genetic programming is a stochastic search approaches, 30 runs have been considered to achieve good statistical confidence. The evolved population contains 1,000 initial and valid strategies generated with the ramped half-and-half algorithm which is a standard in genetic programming. Note that each strategy stops growing once the depth limits is reached in order to avoid the so-called “bloating phenomenon” inherent to all genetic programming approaches having variable-length genome, i.e., an abstract syntax tree as genome representation. The crossover operator is applied between two elite strategies, i.e., the fittest strategies obtained after tournament selection. Finally, the mutation operator which should inject diversity into the population has a low probability to guarantee the convergence of the algorithm.

**Table 4:** Simulation parameters.

**Parameters**	**Training**	**Validation**
Cashflows frequency	Quarterly	Quarterly
Investment period	26 years	26 years
Funds per recommitment	4	4
Fund selection	ESG score	ESG score
Number of simulated portfolios (per evaluation)	250	1,000
Distributed evaluation	True	False

### 5.2. Results discussion

**Figure 9:**
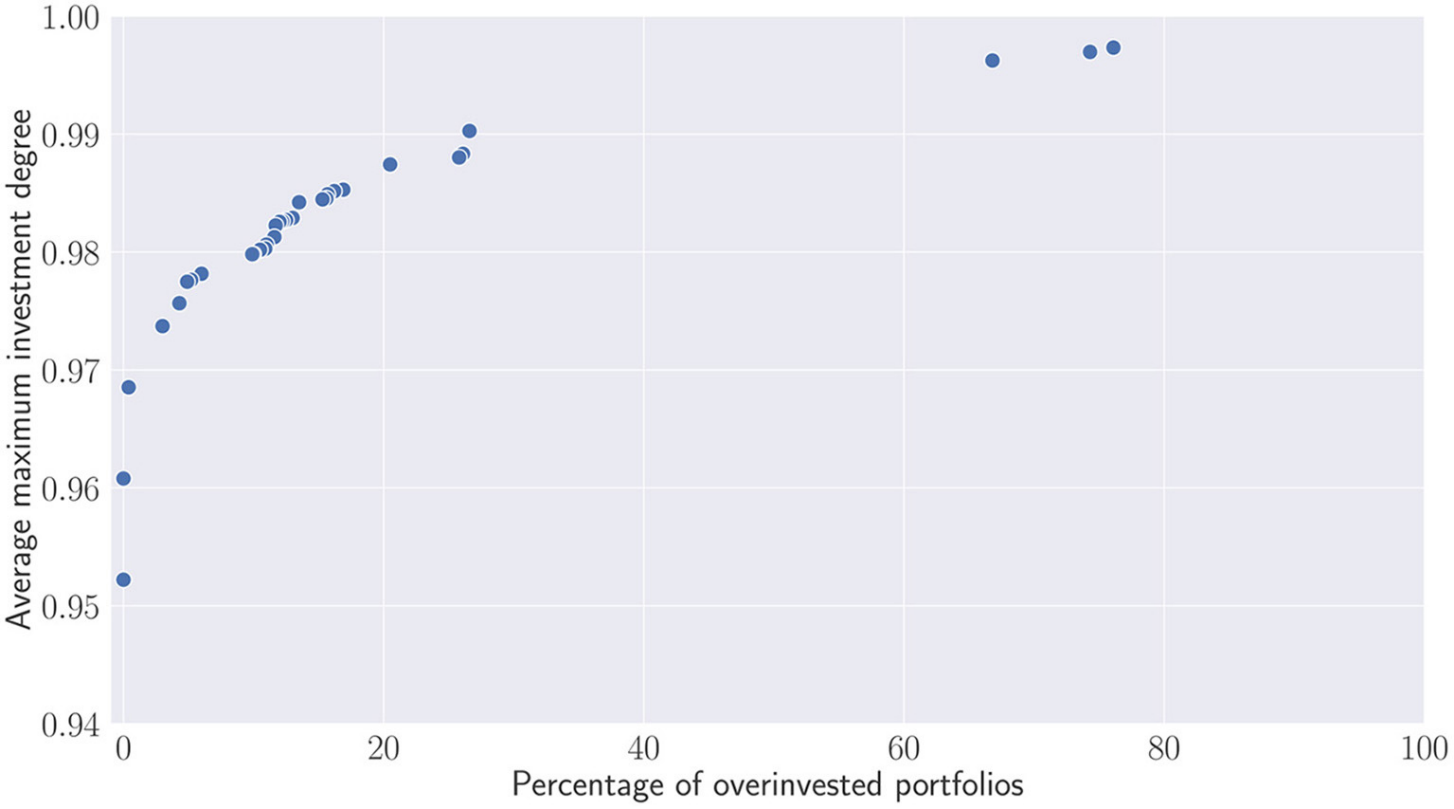
Pareto set of recommitment strategies. Average maximum investment degree V.S. percentage of overinvested portfolios obtained after simulations.

With respect to the parameter described in the previous section, the recommitment strategies obtained for all runs have been merged and a dominance operator have been applied to eliminated dominated strategies. Figure 9 illustrates a Pareto front in which dominated strategies in terms of investment degree and percentage of simulated portfolios becoming overinvested have been removed. On the y-axis, the first metric represents more precisely the average maximum investment degree, i.e., the average maximum value reached by each simulated portfolio during the active recommitment period. On the x-axis, the second metric illustrates the percentage of overinvested portfolios, i.e., portfolios in which additional capital has been injected to satisfy capital calls. Both metrics are clearly conflicting. Recommitment strategies leading to a high maximal investment degree are more likely to yield situations in which portfolios become overinvested. Nonetheless, one can notice that the majority of the strategies generates less than 20% of overinvested portfolios during simulation. The three first strategies represented by the Pareto solutions (0.95; 0), (0.96; 0) and (0.97, 0.4) demonstrate that alternatives leading to no or very few overinvestments are possible as well. Above 0.97, the number of overinvested portfolios steadily increases until reaching 20%. Any attempts to bring the investment degree above 0.99 will result to a large proportion of overinvested portfolios.

**Figure 10:**
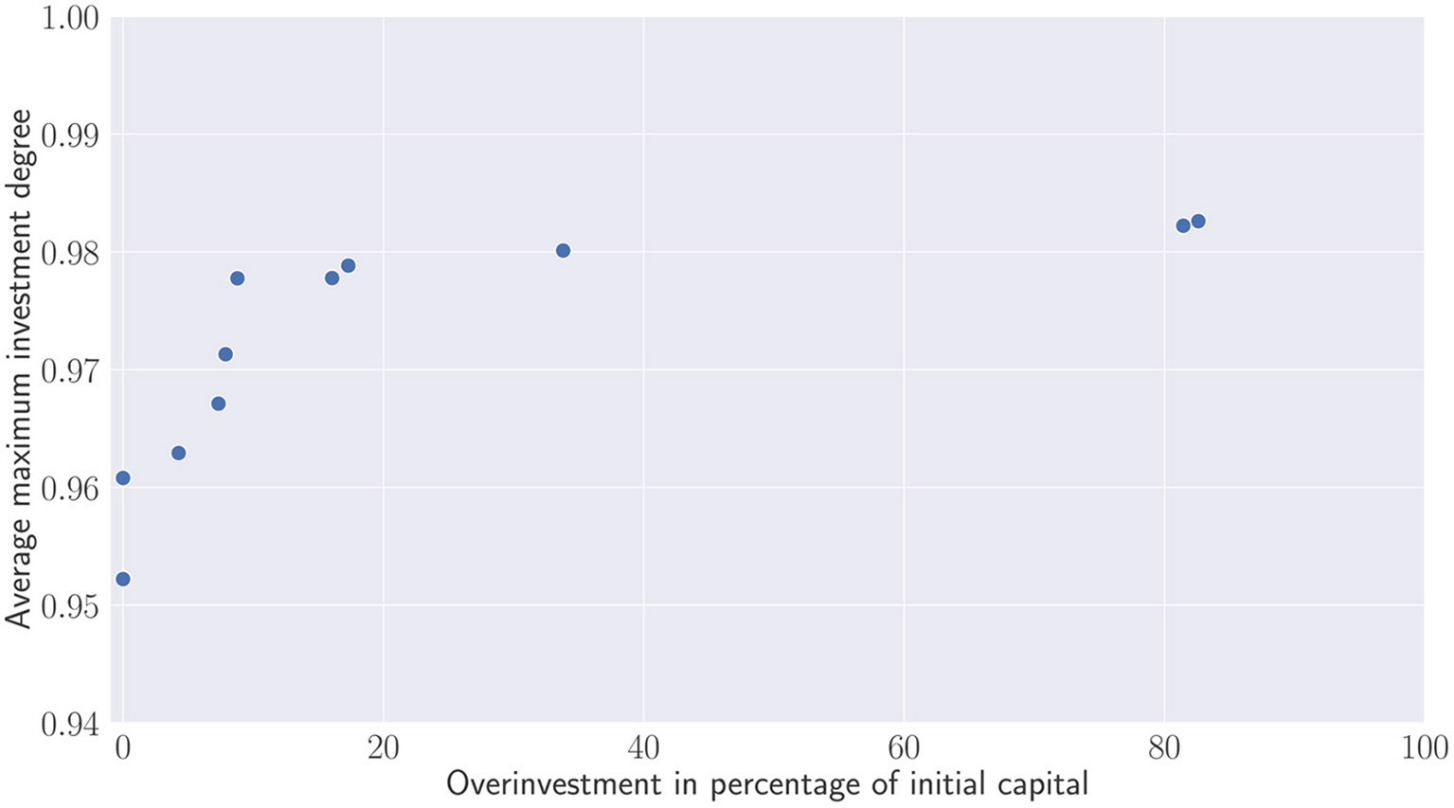
Pareto set of recommitment strategies. Average maximum investment degree V.S. Overinvestment.

Contrary to Figure 9 which evaluate the percentage of overinvested portfolios, Figure 10 depicts the additional capital which has been injected to the overinvested portfolios. This capital is compared relatively to the initial committed capital. Except the two outliers that require re-injecting almost the same capital than the initial one, most of the recommitment strategies lead to less than 20% of overinvestment. The question is now for the investors to find a trade-off and wonder whether the small increases of the investment degree are worth the additional capital used to satisfy capital calls.

These results should be nevertheless compared with the three proposed strategies implemented in de Zwart et al. (2012) which are defined as follows:

-
$DZ^{1}\left(t\right)=D_{t}$

-
$DZ^{2}\left(t\right)=D_{t}+UC_{t-\;24}$

-
$DZ^{3}\left(t\right)=\frac{1}{ID_{t}}\left(D_{t}+UC_{t-24}\;\right)$

*DZ*^1^(*t*) only recommits the distributions received during the current period *t*. *DZ*^2^(*t*) enhances the first proposal by adding the committed but uncalled capital for commitments realized 24 quarters ago. This recommitment strategy relies on the fact that the uncalled capital for funds being in their divestment phase is unlikely to be called and can be therefore recommitted. Finally, the last recommitment strategy *DZ*^3^(*t*) scales the second strategies *DZ*^2^(*t*) by the inverse of the current investment degree. This factor controls the amplitude of recommitment which depends on the current investment degree level. The authors also proposed variants of *DZ*^3^(*t*) taking a desired overcommitment value into account and defined as follows: $DZ_{OC}^{3}\left(t\right)=\frac{1+OC_{t}}{ID_{t}}\left(D_{t}+UC_{t-24}\right)$ with *OC*_*t*_ representing the desired overcommitment applied at each period *t* contrary to the first three strategies in which only an initial and unique overcommitment of 30% has been applied in their simulations.

**Figure 11:**
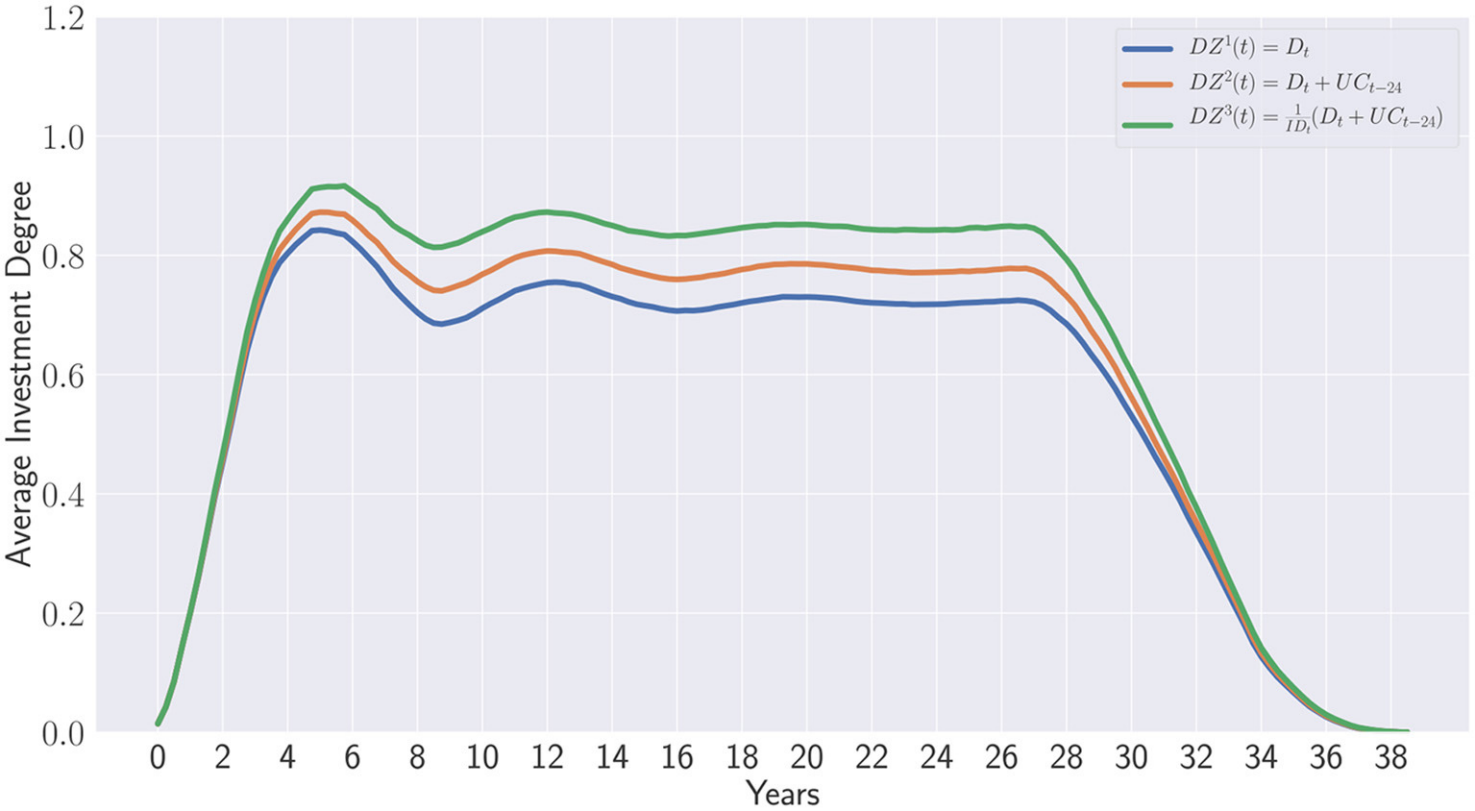
Simulation of the recommitment strategies described in de de Zwart et al. (2012).

Figure 11 illustrates the average investment degrees obtained after applying the three DZ strategies with regards to the data considered in this work. We clearly observed the same trends reported by the original authors. Indeed, the *DZ*^3^(*t*) strategy outperforms clearly the two other strategies *DZ*^1^(*t*) and *DZ*^2^(*t*). Besides, these two strategies do not perform differently as observed in their paper. In fact, the similarities with the original paper confirm the suitability of synthetic cashflow generation.

Please note that we did not discard the first 3 years of the portfolio life's time as done in de Zwart et al. (2012) to observe the influence of the initial overcommitment which clearly improves the investment degree during the 1st year but finally drops and converges to a theoretical value on the long term. All three strategies have a pseudo-periodic regime which is essentially due to this initial overcommitment. Once its effect vanished, one can observed a depreciation of the investment degree which seems to reach a target convergence value.

To counterbalance this problem, the variant $DZ_{OC}^{3}\left(t\right)$ allows investors to overcommit at each period by adapting constantly the overcommitment level. Although this is very appropriate for coping with the depreciation effect observed with the first three strategies, a new question arises: “How much overcommitment should be applied at each period?”.

Finally, the overcommitment applied at each period is difficult to be interpreted relatively to initial committed capital.

### 5.3. Advantages of learning private equity recommitment strategies

**Figure 12:**
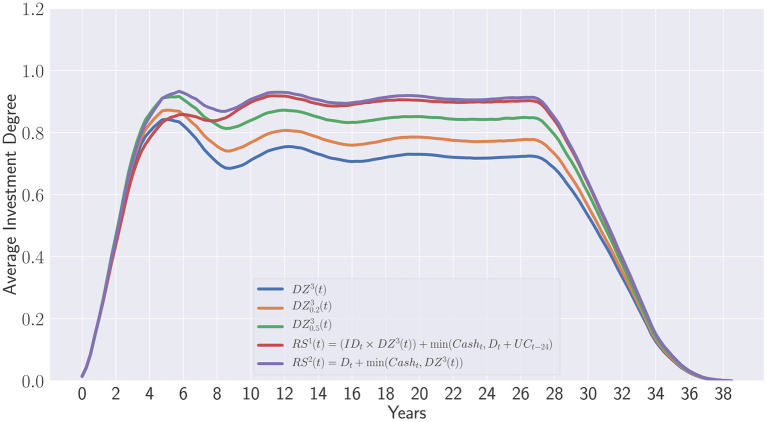
Static overcommitment vs. Dynamic learning.

The approach developed in this work has the benefit to generate new strategies built on top of the seminal work of de Zwart et al. (2012). Instead of defining explicitly a static level of overcommitment at each period, the recommitment strategies are generated and optimized to maximize the investment degree while minimizing the overall overcommitted capital. Figure 12 emphasis the difference between overcommitments in de Zwart et al. (2012) and evolutionary learning of recommitment strategies. The following curves represent the average investment degree obtained during portfolios' lifetime:

- *DZ*^3^(*t*) is the third de Zwart's strategy
- $DZ_{0.2}^{3}\left(t\right)$ is the variant with 20% of periodic overcommitment
- $DZ_{0.5}^{3}\left(t\right)$ is the variant with 50% of periodic overcommitment
-
$RS^{1}\left(t\right)=\left(ID_{t}\times DZ^{3}\left(t\right)\right)+\min\left(Cash_{t},D_{t}+UC_{t-24}\;\right)$

-
${RS^{2}\left(t\right)=D}_{t}+\min\left(Cash<uscore>t,DZˆ3\left(t\right)\right)$

The two last strategies have been obtained in this work and correspond to the points (0.96; 0.0) and (0.98; 0.33) in the Pareto set displayed in Figure 10. Please note that both contain in their respective formulation the last strategy of de Zwart as terminal feature. Although, the strategy *RS*^1^(*t*) does not lead to any additional capital provided by investors, it clearly outperforms all de Zwart's variants relying on static overcommitments on the long term. Interestingly, the strategy *RS*^2^(*t*) only differs from *RS*^1^(*t*) at the beginning of the portfolio lifetime. This is due to the initial overcommitment of 30% which has been applied to each portfolio whatever the recommitment strategy considered for fairness reason. *RS*^2^(*t*) does not seem to be impacted by the initial overcommitment while *RS*^1^(*t*) clearly is. When the influence from the initial portfolio formation period ends, both *RS*^1^(*t*) and *RS*^2^(*t*) become equivalent strategies.

**Figure 13:**
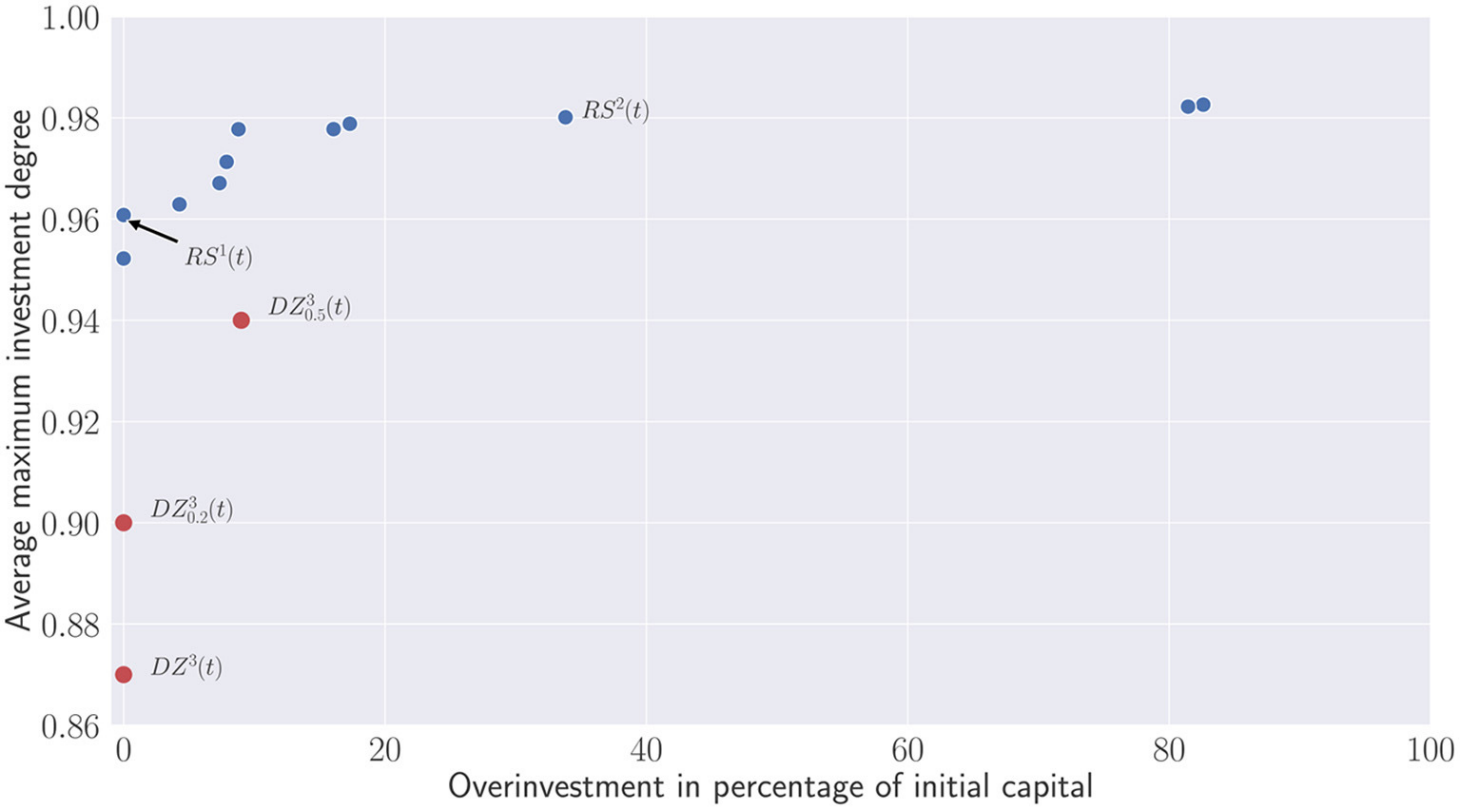
Comparison between de Zwart's variants and the obtained Pareto set.

Finally, one can observe that de Zwart's variants are dominated in terms of Average maximum investment degree and overcommitment in percentage of the initial capital (see Figure 13). This clearly shows that the static overcommitment pacing does not reflect the additional capital injected by investors during the portfolio's lifetime. Despite a 50% overcommitment applied at each period, $D_{0.5}^{3}\left(t\right)$ only represents approximately 9% of the initial capital.

On the contrary, the evolution of the recommitment strategies as described in this work provides a real insight on how much capital should be really re-injected to portfolios to maintain a target exposure.

With regards to the results obtained in this work, further investigations will target multi-asset portfolios to take advantage of different liquidity level and optimize portfolios. Finally, this work was part of a larger project to help investors including ESG considerations. Adding novel criterions will definitely complicate recommitments which should deal with even more objectives. We believe that the automatic of evolution of recommitment strategies could be of great help for investors to maximize their allocation to ESG.

## 6. Conclusion

Discovering efficient recommitment strategies is a real challenge for LP investors who need to develop efficient recommitment mechanisms in order to be kept exposed at a desired level. Although PE has become very popular nowadays, very few academic investigations have been performed to propose more efficient alternatives to the existing recommitment strategies implemented by LPs. In addition, as of today, very few investigations have been conducted to propose an alternative to the *ad-hoc* rules currently in place. They often rely on cashflow forecasting which consisting in predicting next capital calls and distributions. Nonetheless, a new methodology adopting a more pragmatical point of view has emerged. Instead of forecasting data from past cashflows, dynamic recommitment rules or strategies have been proposed in de Zwart et al. (2012) and Oberli (2015). Although, these two contributions offer a real alternative to classical recommitment scheme, they still lack flexibility and could be optimized to provide better proximity to the target exposure. Furthermore, they have been developed using specific cashflow data and may be sub-optimal for different market conditions.

In this work, we proposed to learn these recommitment strategies automatically using a bio-inspired algorithm. Referred to as “evolutionary learning”, a genetic programming algorithm assemble recommitment strategies based on an abstract syntax tree representation. This algorithm relies on Darwin's Theory of Evolution to mimic natural selection by yielding generation after generation novel and promising strategies ensuring efficient recommitment at each period. Using a bi-objective approach, a Pareto set of recommitment strategies had been generated and compared against the seminal work of de Zwart et al. (2012). Empirical results obtained using intensive simulations have shown that the maximum average investment degree can be greatly maximized while providing different alternatives in terms of overcommitment related to the initial capital. Contrary to the static overcommitment seen so far, the strategies obtained by Evolution and validated through simulations provide a real insight in terms of additional capital injected to portfolios during their lifetime. Static overcommitments applied at each period are quite difficult to measure in terms of initial capital and does not reflect the real capital that is actually re-injected.

Further investigations will be performed with multi-asset portfolios to take advantage of the different level of liquidity brought by public market assets.

## Data availability statement

The source code is publicly available online at https://github.com/ekieffer/stairs-code including the synthetic cashflows considered for all the numerical experiments. The authors can also provide more information upon request.

## Author contributions

All authors listed have made a substantial, direct, and intellectual contribution to the work and approved it for publication.

## Footnotes

^1^Such as infrastructure, real estate, forestry and farmland, energy and commodities, and copyrights.

^2^Between 2001 and 2005 this paper had been downloaded more than 10,000 times from Yale School of Management International Center for Finance's website.

^3^The technology is well documented. Since the early 2000s agencies such as Fitch, Moody's, Standard & Poor's, DBRS have been utilizing data from publicly available sources of information on private equity for rating debt backed by interests in a portfolio of funds. These rating agencies initially bought cash-flow data from Venture Economics (now defunct) and in recent years mainly from Burgiss and Cambridge Associates. The rating methodologies are very conservative and apparently the available data is biased towards the universe of institutional quality funds.

^4^Example for monthly periodicity: first payment in February, twice a year.

^5^Real NAVs tend to underestimate the true (unobservable) economic value, as they are appraisal-based valuations with a conservative bias.

^6^For both exhibits simulations were done for fee schedules that spread payments equally over all periods and yields of zero, therefore the results are the same for contributions and drawn-downs and for distributions and repayments, respectively.
